# Chemical Profiling and Antimicrobial Prospective of Commercial Essential Oils from Selected Lamiaceae Genera

**DOI:** 10.3390/molecules31193467

**Published:** 2026-09-29

**Authors:** Alp Giray Akdemir, Kübra Erkan Türkmen, Betül Demirci, Hikmet Katircioglu, Fatma Sezer Şenol Deniz, Ilkay Erdogan Orhan

**Affiliations:** 1 Department of Pharmacognosy, Institute of Health Sciences, Gazi University, 06680 Ankara, Türkiye; 22851702501@gazi.edu.tr; 2 Department of Biology, Faculty of Science, Karamanoglu Mehmetbey University, 70100 Karaman, Türkiye; kerkan@kmu.edu.tr; 3 Department of Pharmacognosy, Faculty of Pharmacy, Anadolu University, 26470 Eskisehir, Türkiye; bdemirca@anadolu.edu.tr; 4 Department of Biology, Faculty of Education, Gazi University, 06560 Ankara, Türkiye; hturk@gazi.edu.tr; 5 Department of Pharmacognosy, Faculty of Pharmacy, Gazi University, 06330 Ankara, Türkiye; fssenol@gazi.edu.tr; 6 Department of Pharmacognosy, Faculty of Pharmacy, Lokman Hekim University, 06510 Ankara, Türkiye

**Keywords:** Lamiaceae essential oils, antimicrobial activity, biofilm inhibition, chemometric analysis, GC-MS profiling, carvacrol and thymol chemotypes, commercial essential oils

## Abstract

Essential oils (EOs) of Lamiaceae family have been reported to possess antimicrobial properties. However, commercial preparations of EOs are often of variable quality and inconsistent efficacy due to adulteration and chemotypic variation. This study comprehensively profiled six retail Lamiaceae EOs (*Origanum vulgare*, *O. vulgare* subsp. *hirtum*, *Thymus vulgaris*, *Lavandula angustifolia*, *Salvia officinalis*, and *Melissa officinalis*) available in Türkiye using GC-MS, complemented by principal component analysis (PCA) and hierarchical cluster analysis (HCA) to discern chemotypic relationships. Antimicrobial and anti-biofilm activities were evaluated against standard bacterial and fungal strains via disc diffusion, broth microdilution, and crystal violet assays. GC-MS and chemometric analyses resolved three distinct chemotypes: phenol-rich (carvacrol/thymol, 49.7–78.2%), ester/alcohol-rich (linalool/linalyl acetate, ~74%), and ketonic/aldehydic-rich (thujone/camphor or citral, 38–54%). Phenol-dominant EOs exhibited potent broad-spectrum activity, with MIC values as low as 0.19% *v*/*v* against *Enterococcus faecalis* and 0.78% *v*/*v* against *Candida albicans*, often surpassing reference antibiotics against Gram-positive and fungal pathogens. In contrast, non-phenolic EOs showed selective or reduced bioactivity, and *Pseudomonas aeruginosa* remained highly resistant across all samples. These findings confirm that retail Lamiaceae EOs retain authentic phytochemical profiles that emphasize their robust, constituent-dependent antimicrobial efficacy, supporting their therapeutic reliability for consumers when quality is assured.

## 1. Introduction

In recent years, the relentless rise in antimicrobial resistance has severely limited the efficacy of conventional synthetic antibiotics and antifungals, prompting an urgent global search for alternative bioactive agents. Plant-derived essential oils (EOs) have re-emerged as premier natural candidate agents due to their exceptionally strong, broad-spectrum antimicrobial properties [1]. Unlike single-target synthetic pharmaceuticals, EOs owe their therapeutic efficacy to their structural diversity and chemical complexity, comprising multi-component mixtures of monoterpenes, sesquiterpenes, aldehydes, and phenolic compounds. They have been reported to exert marked antimicrobial effects through multiple synergistic mechanisms, such as disrupting lipid membranes, altering cell permeability, and inhibiting critical intracellular metabolic pathways, thereby significantly reducing the likelihood of pathogens developing resistance [2,3,4].

Among medicinal and aromatic plant families, Lamiaceae (formerly Labiatae) stands out as one of the most pharmaceutically and commercially valuable biosources of EOs. Several typical genera of Lamiaceae, such as *Origanum*, *Thymus*, *Lavandula*, *Salvia*, and *Melissa,* are widely recognized for their strong biological activities, including antimicrobial effects as well as their use as spices [5,6,7,8]. While the broad bioactivity profiles of these botanical taxa are well-documented in the literature, a significant gap remains regarding the quality, chemical consistency, and biological potency of commercially available EOs. EOs of commercial origin, widely distributed to consumers and therapeutic sectors, often display considerable variations in their chemical profiles due to factors such as geographical origin, harvesting season, extraction methodologies, and post-harvest handling. Beyond environmental and post-harvest variations, the commercial EO market faces widespread chemical adulteration. Driven by high consumer demand, economic incentives, and limited regulatory oversight, commercial EOs are frequently subjected to synthetic spiking, dilution with cheap carrier oils or low-cost volatile fractions, or mislabeling of botanical sources [9,10,11]. Such adulteration in EOs considerably alters the natural synergy of multi-component matrices, compromising therapeutic consistency, reducing antimicrobial efficacy, and potentially introducing toxic or allergenic synthetic contaminants [12,13].

Herbal products range from local to international commercial goods. Assessing commercially available pharmaceutical/medicinal preparations of herbal origin before and after being released to the market is therefore of vital importance to verify their chemical composition for both quality assurance and consumer safety [14]. In this context, obtaining commercial EOs specifically from pharmacies provides a vital quality-assurance advantage; unlike unregulated retail outlets, pharmacies operate under strict legal frameworks, routine regulatory monitoring, and proper depot storage conditions (such as controlled temperature and light protection) that protect volatile constituents from thermo-oxidative degradation. While the chemical composition and antimicrobial properties of individual Lamiaceae EOs have been the subject of extensive investigation, the vast majority of these studies have focused on laboratory-distilled EOs obtained from plant materials of known provenance under controlled extraction conditions. Consequently, critical knowledge gaps remain regarding: (i) whether commercially available, pharmacy-purchased EOs retain their expected phytochemical authenticity and biological potency; and (ii) how EOs from different Lamiaceae genera compare directly under standardized experimental conditions. Multi-genus comparative studies that integrate chemometric profiling with quantitative antimicrobial and antibiofilm evaluation of commercial retail products are notably scarce. The present study was therefore designed to address these gaps by providing a comprehensive, side-by-side comparative analysis of six pharmacy-purchased Lamiaceae EOs, thereby generating new insights into the relationship between chemotypic composition and antimicrobial efficacy in products that consumers actually use.

Consequently, in the current study, we performed a comprehensive chemical profiling using gas chromatography-mass spectrometry (GC-MS) as well as unsupervised chemometric techniques such as principal component analysis (PCA) and hierarchical cluster analysis (HCA) of commercially available Lamiaceae EOs (*Origanum vulgare* L./OV, *Origanum vulgare* subsp. *hirtum* (Link) Ietsw./OVH, *Thymus vulgaris* L./TV, *Lavandula angustifolia* Mill./LA, *Salvia officinalis* L./SO, and *Melissa officinalis* L./MO), purchased from community pharmacies in Türkiye, as well as their comparative antimicrobial and anti-biofilm evaluation. By evaluating retail preparations against standard strains of the key bacterial (*Pseudomonas aeruginosa* PAO1, *Escherichia coli* ATCC 25922, *Enterococcus faecalis* ATCC 29212, and *Staphylococcus aureus* ATCC 29213) and fungal (*Candida albicans* ATCC 10231) pathogens, we sought to verify whether off-the-shelf commercial products retain their expected phytochemical authenticity and high-potency antimicrobial efficacy.

## 2. Results

### 2.1. GC-MS Profiling of the EOs

The chemical compositions of the evaluated retail EOs belonging to the Lamiaceae family were identified using GC-MS analysis, with total identified compound proportions ranging from 90.6% to 99.9% (Table 1) and their corresponding GC chromatograms (Figure 1). According to our data, OVH was characterized as a strong carvacrol chemotype, containing carvacrol (78.0%) as the primary volatile constituent, along with precursor monoterpenes *p*-cymene (7.5%) and γ-terpinene (5.3%), while OV was similarly dominated by carvacrol (72.6%), supported by linalool (6.6%) and *p*-cymene (5.1%). The commercial sample of TV was defined as a thymol chemotype, with major components identified as thymol (48.1%), *p*-cymene (24.2%), and γ-terpinene (16.7%). LA displayed a similar volatile profile comprising linalool (37.2%) and linalyl acetate (36.1%), with camphor (3.8%), 1,8-cineole (3.9%), and borneol (2.6%).

The chemical functional group classification reveals clear phytochemical clustering among the evaluated retail Lamiaceae EOs, which directly accounts for their varying biological potencies (Table 2). The commercial preparations fall into three distinct profiles: phenol-dominant oils (OVH, OV, and TV), which possess high concentrations of active phenolics (49.7–78.2%; carvacrol and thymol) alongside precursor monoterpene hydrocarbons (*p*-cymene and γ-terpinene); ketonic/aldehydic-rich oils (SO and MO), characterized by elevated ketones (54.6%; α-thujone and camphor) or aliphatic aldehydes (38.2%; geranial, neral, and citronellal) paired with a high sesquiterpenic fraction (42.6% total in MO); and ester/alcohol-rich oils (LA), which feature a balanced monoterpenoid matrix dominated by linalool (40.9% total alcohol) and linalyl acetate (39.5% total ester) with negligible phenolic content. This structural division directly correlates with the strong, broad-spectrum antimicrobial efficacy exhibited by the carvacrol- and thymol-rich samples compared to the target-specific bioactivity observed in the non-phenolic EOs.

### 2.2. Antimicrobial Activity Data of the EO Samples

The outcomes obtained from the disc diffusion method and minimum inhibitory concentration (MIC) determination are tabulated in Table 3 and Table 4 as well as Figure 2. Accordingly, the phenol-rich EOs, OVH, OV, and TV, exhibited pronounced antibacterial activity against *E. coli*, each producing inhibition zones of over 55 mm (55.33 ± 0.58 mm for OVH and OV, 55.67 ± 0.58 mm for TV). These EOs significantly outperformed the reference antibiotic ciprofloxacin (34.33 ± 0.58 mm). Conversely, LA showed moderate activity (16.67 ± 0.58 mm), SO displayed weak activity (9.67 ± 0.58 mm), and MO demonstrated no inhibition. Considering *E. faecalis* ATCC 29212, OVH (89.33 ± 0.58 mm) and TV (88.67 ± 0.58 mm) demonstrated the highest potency, nearly doubling the inhibition zone of the reference drug ampicillin (48.33 ± 0.58 mm). OV also surpassed ampicillin with a zone of 63.67 ± 0.58 mm. MO displayed moderate activity (38.33 ± 0.58 mm), while LA (19.33 ± 0.58 mm) and SO (12.67 ± 1.15 mm) were considerably less effective than the reference standard. TV (72.33 ± 0.58 mm), OVH (60.67 ± 1.15 mm), and OV (50.67 ± 0.58 mm) all exceeded the efficacy of rifampin employed as the reference antibiotic (45.33 ± 0.58 mm) against *S. aureus*. LA and MO displayed moderate activity (20.33 ± 0.58 mm and 20.66 ± 0.58 mm, respectively), whereas SO produced weak inhibition (10.67 ± 1.15 mm). Towards *P. aeruginosa*, all evaluated EOs yielded modest inhibition zones ranging from 10.33 ± 0.58 mm (SO) to 16.67 ± 0.58 mm (OV). None of the EOs approached the efficacy of ciprofloxacin (50.33 ± 0.58 mm), highlighting the resistance of this Gram-negative strain.

For the antifungal activity results against the fungal strain (*C. albicans*), TV (74.33 ± 0.58 mm), OVH (61.33 ± 1.53 mm), MO (55.67 ± 0.58 mm), and OV (52.67 ± 0.58 mm) exhibited marked antifungal activity, markedly surpassing the reference antifungal agent amphotericin B (22.67 ± 0.58 mm). LA (14.67 ± 1.15 mm) and SO (11.33 ± 0.58 mm) produced lower activity relative to amphotericin B.

The two-way ANOVA showed that inhibition-zone diameters were significantly influenced by the EO type, microbial strain, and their interaction (*p* < 0.0001). As shown in Figure 2, OVH, TV, and OV generally produced the largest inhibition zones, particularly against *E. faecalis*, *S. aureus*, *E. coli*, and *C. albicans*, demonstrating broad-spectrum antimicrobial activity. The strong activity of these oils may be associated with their high carvacrol and thymol contents. In contrast, *P. aeruginosa* exhibited comparatively low susceptibility to all EO samples, with inhibition zones ranging from 10.33 to 16.67 mm. MO displayed a selective antimicrobial profile, producing substantial inhibition zones against *C. albicans* and *E. faecalis*, moderate activity against *S. aureus*, and no detectable activity against *E. coli*.

The MIC values presented in Table 4 further confirm the potent, strain-dependent bioactivity observed in the disc diffusion screen. The phenol-rich EO samples, namely OVH, OV, and TV, exhibited the strongest overall antimicrobial efficacy, displaying remarkably low MIC values across both Gram-positive bacteria and *C. albicans*. TV demonstrated the highest intrinsic potency among all tested EOs, suppressing the growth of *E. faecalis* at a concentration as low as 0.19% *v*/*v*, while recording an MIC of 0.78% *v*/*v* against both *S. aureus* and *C. albicans*. OVH and OV also displayed robust activity with MICs ranging between 0.39% and 1.56% *v*/*v* for these susceptible strains. In contrast, non-phenolic EOs required higher concentrations to inhibit microbial growth. LA and SO yielded higher MIC values overall, generally requiring 3.12% to 6.25% *v*/*v* to achieve total growth inhibition. Notably, MO exhibited selective potency; while it was inactive against *E. coli*, it demonstrated strong antifungal activity against *C. albicans* with an MIC of 1.56% (*v*/*v*), matching the potency of the carvacrol-rich *Origanum* oils. Across all tested samples, *P. aeruginosa* displayed the highest tolerance, requiring elevated MIC values (3.12% to 6.25%, *v*/*v*), which reinforces the intrinsic resistance of this Gram-negative pathogen to volatile terpene matrices.

In addition, a biofilm inhibition assay was carried out with the retail EO samples. As presented in Table 5 and Figure 3, the crystal violet assay revealed that the retail EO samples exhibited concentration-dependent anti-biofilm activities. The phenol-rich OVH, OV, and TV samples demonstrated the highest efficacy, achieving pronounced biofilm inhibition against *E. faecalis* (83.67% to 84.67%) and moderate-to-high reductions across *E. coli*, *S. aureus*, and *C. albicans* biofilms. This strong antibiofilm performance is attributed to the membrane-disrupting and permeability-altering properties of carvacrol and thymol, which effectively impair the structural integrity of the extracellular polymeric substance (EPS) matrix. Conversely, the non-phenolic EOs (LA and SO) displayed considerably lower antibiofilm activity, generally suppressing biofilm biomass by under 35%. Notably, MO exhibited a selective bioactivity trend, showing prominent inhibition against *C. albicans* biofilms (58.33%) while proving ineffective against *E. coli* planktonic and biofilm growth. Overall, biofilms formed by *P. aeruginosa* were less susceptible to the most active EO samples than those formed by the other tested microorganisms.

### 2.3. Chemometric and Multivariate Analysis Data

To further evaluate the phytochemical interrelationships and chemotypic discrimination among the commercial Lamiaceae EOs, unsupervised principal component analysis (PCA) and hierarchical cluster analysis (HCA) integrated with a heatmap were performed based on the major volatile constituents (≥3.0%). The PCA score plot (Figure 4) accounted for over 85% of the total cumulative variance across the first two principal components. PC1 cleanly differentiated the phenol-rich essential oils (OVH, OV, and TV) from the non-phenolic formulations, driving them into distinct clusters enclosed by 95% confidence ellipses. OVH and OV grouped tightly on the positive PC1 domain due to their dominant carvacrol content, while TV positioned adjacent based on its high thymol and *p*-cymene. Conversely, PC2 separated the non-phenolic EOs based on their specific monoterpenoid and sesquiterpenoid profiles, placing LA in the upper quadrant due to its high proportion of linalool and linalyl acetate, while SO and MO occupied the lower quadrants defined by elevated ketone (α-thujone, camphor), aldehyde (geranial, neral), and sesquiterpene (β-caryophyllene, caryophyllene oxide) fractions.

The two-way HCA heatmap (Figure 5) corroborated the spatial clustering observed in the PCA model, grouping both the volatile compounds and EO samples using Ward’s minimum variance method. The column dendrogram separated the six retail preparations into three primary bioactivity clades corresponding directly to the top annotation track: a phenol-rich cluster (OVH, OV, and TV), an ester/alcohol cluster (LA), and a carbonyl/sesquiterpenoid cluster (SO and MO). The row dendrogram highlights distinct compound modules that reveal these chemotypes, clearly illustrating how the high relative abundance of active phenolic monoterpenes in OVH, OV, and TV samples underpins their superior broad-spectrum antimicrobial potency compared to the specialized profiles of the non-phenolic retail samples. When interpreted alongside antimicrobial bioassays, the two-way HCA heatmap visually demonstrates the structure-activity relationships of the evaluated EO samples. The intense red clusters corresponding to phenolic monoterpenes (carvacrol and thymol) and their monoterpene hydrocarbon precursors (*p*-cymene and γ-terpinene) serve as direct markers of high broad-spectrum antibacterial and antifungal potency. In contrast, the distinct aldehyde module (geranial/neral) highlighted in red for MO strengthens its target-specific antifungal efficacy against *C. albicans*, while the overall absence of phenolic red spots in LA and SO accounts for their comparatively lower membrane-disrupting capacity.

## 3. Discussion

Detecting adulteration in EOs is a critical challenge due to the economic incentive to stretch high-value botanical oils with cheaper synthetic alternatives, solvents, or lower-grade plant extracts. While GC-MS remains the traditional benchmark for volatile terpenes, authenticating EOs effectively requires matching the technique to the target using Fourier transform infrared spectroscopy (FT-IR) and ambient MS for ultra-fast screening, quantitative nuclear magnetic resonance spectroscopy (qNMR) to capture non-volatile adulterants and structure, and gas chromatography–combustion–isotope ratio mass spectrometry (GC-C-IRMS) to conclusively distinguish natural compounds from synthetic additions [9,10,11,15,16]. The choice of analytical technique depends heavily on the specific target analyte, the operational constraints of the testing facility, and whether the primary goal is high-throughput screening or definitive structural verification. In this regard, gas chromatography equipped with a flame ion detector (FID) or MS is considered the benchmark standard across International Organization for Standardization (ISO) frameworks and pharmacopeias for terpene profiling, component quantification, and mass spectral fingerprinting [13,17,18,19]. It offers exceptionally high separation efficiency, detailed volatile identification, and chiral separation capabilities to evaluate enantiomeric ratios. Our GC-MS profiling of the retail EO samples demonstrated that their volatile compositions align closely with literature values and established pharmacopeial standards regarding key characteristic constituents (Table 1 and Table 2, Figure 1). These findings suggest that the evaluated commercial preparations retain their expected phytochemical profiles and appear suitable for therapeutic or consumer applications. In other words, although standard GC-MS cannot rule out sophisticated synthetic spiking, the observed volatile profiles suggest that these commercial products retain authentic chemotypic characteristics appropriate for consumer use. A comparative evaluation of the main volatile constituents of the commercial Lamiaceae EOs against established literature data and international standards (ISO and European Pharmacopoeia) confirms their phytochemical integrity. GC-MS chemical composition studies reveal that EOs from OV are predominantly dominated by oxygenated monoterpenes and their biosynthetic precursors. The carvacrol dominance in OVH (78.0%) and OV (72.6%), together with the thymol level in TV (48.1%), reflects the typical profile via the cymyl pathway of monoterpenene biosynthesis of Mediterranean populations, distinguishing them from acyclic (linalool/linalyl acetate) wild OV chemotypes [20,21,22,23,24]. Our findings for OVH (78.0% carvacrol) align closely with the cymyl-pathway chemotype cluster reported by recent studies on Mediterranean OV populations. While wild OV accessions exhibit considerable intraspecific variation, including distinct acyclic profiles dominated by linalyl acetate and linalool, our OVH sample demonstrated the characteristic phenolic monoterpene dominance typical of OVH, driven by active biosynthesis along the γ-terpinene—*p*-cymene—carvacrol pathway [25].

Furthermore, our LA retail sample exhibited a balanced linalool-to-linalyl acetate ratio (37.2% and 36.1%, respectively), complying with ISO 3515:2002 specifications as well as the reported data on the same species [26]. Across the relevant studies, EOs from LA are overwhelmingly dominated by oxygenated monoterpenes, specifically linalyl acetate and linalool, though their relative proportions fluctuate based on geographic origin, extraction technique, and plant morphology [27,28,29]. GC-MS data across LA EOs confirmed an oxygenated monoterpene profile dominated by linalool (16.58–51.5%) and linalyl acetate (27.6–51.06%), alongside variable lavandulyl acetate (2.25–23.2%), 1,8-cineole (1.19–4.8%), and low camphor (0.28–0.40%) [30,31,32,33,34].

SO was compliant with the ISO 9909:1997 monograph for α-thujone (33.8%) and camphor (15.0%) [35]. The principal bioactive ketone α-thujone reaches 33.8% (strictly within the ISO standard range of 18.0–43.0%), while camphor stands at 15.0% (ISO standard 4.5–24.5%). Additional characteristic constituents support its authenticity, including 1,8-cineole (8.0%), α-humulene (7.1%), α-pinene (6.8%), β-thujone (5.8%), and β-caryophyllene (3.8%). Our GC-MS data for SO retail EO sample aligns closely with the oxygenated monoterpene- and ketone-rich profiles reported across Mediterranean and European accessions, obviously matching the α-thujone-dominant chemotypes from Spain (22.8–41.7% α-thujone) [36], Romania (31.23–52.86% α-thujone) [37], and Croatia (native Dalmatian populations rich in α/β-thujone, camphor, and 1,8-cineole) [38], while contrasting with camphor/1,8-cineole-dominant profiles observed in Southern Italy (where α-thujone drops to 1.17–9.26%) and certain commercial dried aerial parts of SO [39]. However, a neurotoxicity risk might be available for EOs such as SO containing a marked level of thujone [40,41]. While aqueous infusions (sage tea) remain safe for short-term consumption due to poor water solubility (0.3 mg/cup) of the compound, the high α-thujone level in pure SO retail EO sample (33.8%) presents a severe neurotoxic risk via gamma-aminobutyric acid type A (GABA_A_) receptor antagonism that can rapidly induce epileptic seizures upon direct accidental ingestion, far exceeding the European Pharmacopoeia quality specifications, the European Medicine Agency (EMA) daily limit (5.0 mg/day), and the acceptable daily intake (ADI, 6.6 mg/day), thereby emphasizing the necessity for strict dose management, precautionary labeling, or selective dermal formulations [42,43].

Finally, the combined citral fraction (geranial + neral = 29.6%) and significant sesquiterpenic profile (β-caryophyllene, 16.7%) in MO align closely with the reported benchmarks for commercial lemon balm oils. Across the evaluated geographic regions, EOs from MO display significant chemotypic diversity influenced by origin, maturity, and processing methods. *M. officinalis* subsp. *officinalis* profile—dominated by the monoterpene aldehydes geranial and neral (citral)—is consistently observed in Algerian, Polish, Italian, Indian, and Turkish samples, with Turkish (Adana province) and Algerian samples reaching high citral concentrations of 65.48% and 76.78%, respectively [44,45,46,47,48]. In contrast, distinct geographical chemotypes emerge in Romanian samples, which are dominated by sesquiterpenes (β-cubebene at 27.66% and β-caryophyllene at 27.41%), and Moroccan samples, which are characterized by oxygenated monoterpenes like *p*-mentha-1,2,3-triol (13.1%) and pulegone (8.8%) [49,50]. Furthermore, harvesting timing and plant age induce pronounced compositional shifts; for instance, Polish cultivations demonstrate a conversion of citral into citronellal (reaching up to 60.8%) during late autumn harvests [51]. Finally, commercial market samples from Taiwan exhibit substantial batch-to-batch variation and oxidation effects during storage, such as elevated β-caryophyllene oxide levels (up to 31.73%) and geranic acid formation [52]. Collectively, these comparative chromatographic data confirm that the evaluated retail preparations maintain their expected characteristic volatile profiles, aligned closely with the profiles reported in the literature and standard monographs.

The antimicrobial evaluation of commercial Lamiaceae EOs reported in our study demonstrates consistent broad-spectrum efficacy, aligning with findings across relevant literature [53,54,55]. Our retail TV (48.1% thymol) and OV (72.6% carvacrol) samples generated remarkably potent inhibition zones (>50 mm) and low MIC values (0.19–1.56% *v*/*v*) against *E. coli*, *E. faecalis*, *S. aureus*, and *C. albicans*. These findings reflect those of Puškárová et al. from Slovakia [56], who demonstrated that high-phenol OV and TV EOs exerted remarkably strong bactericidal and fungicidal activities against *S. aureus*, *E. coli*, and *E. faecalis* (MICs 0.025–0.125%), even outperforming conventional antibiotics like chloramphenicol. Similarly, in Mexico, Hersch-Martínez et al. reported that commercial EO samples of TV and OV exhibited the broadest antibacterial activity against drug-resistant pediatric clinical isolates, including *S. aureus* (inhibition zones 22.8–27.5 mm) and *E. coli* (18.4–22.2 mm) [57]. The high bioactivity observed in our carvacrol- and thymol-rich EO retail samples aligns with Ahmad et al. from South Africa, who showed that monoterpene phenols (thymol and carvacrol) drive the primary antimicrobial efficacy of TV EO through membrane depolarization and intracellular targeting [58]. Ahmad et al. further noted that precursor monoterpene hydrocarbons like *p*-cymene work synergistically with carvacrol and thymol to enhance cell wall penetration, a mechanism that directly supports the high activity of our *p*-cymene-containing TV (24.2%) and OV (5.1%) samples. Furthermore, Pradebon Brondani et al. from Brazil reported that OV EO significantly reduced *C. albicans* phospholipase exoenzyme production, echoing the strong antifungal inhibition against *C. albicans* observed in our retail OV (52.67 mm) and TV (74.33 mm) samples [59]. When comparing the literature findings for SO, MO, and LA directly with our experimental results, clear chemotypic alignments and bioactivity differences emerge. For SO, our retail sample presented an α-thujone (33.8%) and camphor (15.0%) profile but displayed weak overall antimicrobial activity across all strains (MICs 6.25% *v*/*v*, inhibition zones 9.67–12.67 mm). This weak antimicrobial potency mirrors the observations of Karpiński et al., who reported that SO exhibited the lowest anti-*Candida* activity among Lamiaceae species (MICs 3.125–100 mg/mL) [60], as well as El Jery et al. [61] and Ben Khedher et al. [62], who noted modest antifungal values for sage. While Đurović et al. [63] and El Ouadi et al. [49] demonstrated that specific extraction methods (e.g., high-power hydrodistillation) or postharvest fungal applications can enhance SO performance, our standardized retail preparation confirmed its limited direct membrane-disrupting capacity against clinical and standard pathogens [63]. For MO, our retail sample was defined by a citral/citronellal aldehyde fraction (38.2%) and high sesquiterpenes (28.0%), exhibiting selective, high-potency antifungal activity against *C. albicans* (MIC 1.56% *v*/*v*, 55.67 mm zone). This strongly aligns with Cavalcanti et al., who established the potent anti-yeast action of geranial-rich MO (MIC 26.7 μg/mL against *C. auris*) [64], Karpiński et al., who noted its high planktonic anti-*Candida* potency (MIC < 3.125 mg/mL) [60], and El Ouadi et al., who reported low MICs against agricultural mold pathogens [49]. Finally, for LA, our retail sample possessed a balanced linalool (37.2%) and linalyl acetate (36.1%) profile and exhibited moderate broad-spectrum growth inhibition (MICs 3.12–6.25% *v*/*v*). Our experimental findings sit right between the weak antifungal efficacy (MIC > 10% *v*/*v*) reported by Stamova et al. for specific LA cultivars [65].

The retail EO samples inhibited biofilm formation to markedly different extents, indicating that antibiofilm activity depended on both EO composition and the target microorganism. At ½ MIC, the carvacrol-rich OVH and OV samples and the thymol-rich TV sample showed the greatest overall activity. OVH and TV were particularly effective against *E. faecalis*, reducing biofilm biomass by 84.67% and 83.67%, respectively. TV also produced substantial inhibition against *S. aureus* (70.67%), *C. albicans* (70.33%), and *E. coli* (66.67%). OVH and OV showed similarly broad, although microorganism-dependent, activity. These findings are consistent with the antibiofilm potential previously reported for carvacrol- and thymol-rich Lamiaceae EOs. Zhan et al. demonstrated that oregano EO inhibited *E. faecalis* biofilm development and reduced the viability of biofilm-associated cells, supporting the pronounced activity of OVH and TV against *E. faecalis* observed in the present study [66]. Similarly, a recent investigation of thyme EO reported inhibition of biofilm formation by several Gram-positive and Gram-negative bacteria, including *S. aureus*, *E. coli*, and *P. aeruginosa*, although the magnitude of the response varied according to the microorganism and EO concentration [67].

The strong activity of OVH, OV, and TV may be associated with their high concentrations of carvacrol and thymol. These phenolic monoterpenes can affect membrane integrity, cellular homeostasis, adhesion, motility, quorum-sensing-associated processes, and the production of extracellular matrix components. Current evidence suggests that EO-mediated biofilm inhibition is likely to involve several simultaneous cellular targets rather than a single mechanism [68]. The presence of *p*-cymene and γ-terpinene may also influence the overall activity of phenolic-rich oils through interactions with carvacrol and thymol. Nevertheless, the contribution of individual constituents and possible synergistic interactions were not examined directly in the present study. The association between phenolic monoterpene content and antibiofilm activity should therefore be interpreted as a composition–activity relationship rather than proof of a specific molecular mechanism.

The activity of TV against *C. albicans* was also consistent with previous findings for thyme EO and thymol. Jafri and Ahmad reported that *T. vulgaris* EO and thymol inhibited biofilm development by *C. albicans* and *C. tropicalis* when applied at ½ MIC; microscopy further demonstrated reduced biofilm organization and altered fungal morphology [69]. Karpiński et al. likewise found that oregano and thyme EOs were among the most effective Lamiaceae oils against *Candida* biofilms, although the magnitude of inhibition depended on the EO and *Candida* strain [60]. In the present study, TV caused 70.33% inhibition of *C. albicans* biofilm formation at ½ MIC, whereas OVH and OV produced inhibition values of 64.33% and 58.33%, respectively. These results support the antibiofilm potential of phenolic-rich *Thymus* and *Origanum* oils while also demonstrating that activity against planktonic cells and biofilm formation should be evaluated as distinct biological outcomes.

MO displayed a selective activity profile, inhibiting *C. albicans* biofilm formation by 58.33% but producing no detectable inhibition against *E. coli*. The activity against *C. albicans* may be related to its citral components, geranial and neral, together with citronellal and β-caryophyllene. Ranđelović et al. reported that *M. officinalis* EO inhibited biofilms formed by multidrug-resistant clinical *Candida* isolates, supporting the antifungal biofilm activity observed for the MO sample [70]. However, Karpiński et al. found lemon balm EO to be less effective against *Candida* biofilms than oregano and thyme oils [60]. This is broadly compatible with the present findings because MO inhibited *C. albicans* biofilm formation but remained less active than TV. Differences among studies may reflect variations in chemotype, citral content, fungal strain, exposure concentration, biofilm maturity, and the assay used to quantify biofilm biomass.

LA and SO exhibited comparatively limited antibiofilm activity, with inhibition generally remaining below 35%. Against *C. albicans*, LA and SO reduced biofilm formation by only 27.33% and 19.33%, respectively. This lower activity agrees with the overall composition–activity pattern observed in the present study, in which the oils dominated by linalool/linalyl acetate or α-thujone/camphor were less active than carvacrol- and thymol-rich oils. Karpiński et al. similarly reported a weaker antibiofilm activity for sage EO than for oregano and thyme EOs against *Candida* species [60]. Nevertheless, these findings should not be generalized to all lavender or sage EOs, because variations in botanical origin, chemotype, harvesting conditions, storage, and EO composition may substantially alter biological activity.

Across the most active samples, *P. aeruginosa* biofilm formation was less affected than that of the other tested microorganisms. OVH, OV, and TV produced inhibition values of only 30.67–38.67% against *P. aeruginosa*, compared with 57.33–84.67% against the other bacterial and fungal strains. The comparatively low susceptibility of *P. aeruginosa* may be associated with its restrictive outer membrane, active efflux systems, adaptable metabolism, quorum-sensing networks, and structurally complex biofilm matrix. However, thyme EOs with different chemical profiles have shown variable antibiofilm activity against *P. aeruginosa*. Bakó et al., for example, demonstrated that the harvesting stage and resulting composition of *T. vulgaris* EO substantially affected its activity against *P. aeruginosa* biofilms [71]. Therefore, the lower inhibition observed in the present study should be interpreted as a characteristic of the tested retail samples under the specified experimental conditions rather than as universal resistance of *P. aeruginosa* to Lamiaceae EOs.

On the other hand, the chemical structure and functional group profile of EO constituents directly influence their observed biological activity against the evaluated microbial strains. The exceptionally high antimicrobial activity observed in OVH, OV, and TV is primarily driven by their high concentration of phenolic monoterpenes, specifically carvacrol and thymol [72,73,74]. Phenolic compounds possess an aromatic benzene ring linked to a free hydroxyl (-OH) group [72]. The lipophilic nature of the benzene ring enables these molecules to readily partition into the hydrophobic lipid bilayer of bacterial and fungal cell membranes [74]. Once inserted, the acidic, free hydroxyl group acts as a proton exchanger, collapsing the transmembrane pH gradient and electrical potential. This disruption leads to membrane leakage, rapid depletion of intracellular adenosine triphosphate (ATP), and eventual cell lysis [72]. Furthermore, the presence of hydrocarbon precursor monoterpenes like *p*-cymene and γ-terpinene plays a key synergistic role [72,75]. Lacking polar functional groups, *p*-cymene possesses high lipophilicity but minimal intrinsic antibacterial effect; however, its integration into the membrane causes structural swelling and increases membrane fluidity, thereby facilitating the penetration of phenolic active molecules into the cell. In contrast, EOs dominated by non-phenolic oxygenated monoterpenes exhibit reduced or specialized antimicrobial effects [72,75]. The moderate activity shown by LA is attributed to linalool and linalyl acetate. Linalool contains a polar tertiary alcohol group that lacks the conjugated phenolic system, resulting in weaker membrane-disrupting capabilities. Converting this alcohol into an ester, as in linalyl acetate, lowers water solubility and reduces hydrogen-bonding potential, which diminishes direct interaction with structural membrane proteins. Similarly, the predominantly ketone-based profile of SO, rich in α-thujone and camphor, demonstrates the lowest overall antimicrobial activity. Ketone carbonyl groups (C=O) form considerably weaker hydrogen bonds with cell membrane components compared to free phenolic hydroxyl groups, leading to limited membrane instability. A unique target-specific activity profile is observed with MO, which contains aliphatic aldehydes such as geranial, neral, and citronellal [72]. These aldehyde monoterpenes can cross-link or covalently bind with amine and thiol functional groups on essential membrane proteins and cell wall enzymes. Consequently, MO exhibits pronounced selectivity, demonstrating strong antifungal activity against *C. albicans* and high antibacterial action against Gram-positive *E. faecalis*, while proving completely ineffective against Gram-negative *E. coli*. Finally, the uniformly weak inhibition observed across all EOs against *P. aeruginosa* illustrates a key structural barrier characteristic of certain Gram-negative bacteria [72,74]. The presence of an outer membrane rich in lipopolysaccharides (LPS), combined with active multidrug efflux pump systems, severely restricts the influx of lipophilic monoterpenes regardless of their chemical functional groups, rendering the essential oils significantly less potent than conventional antibiotics like ciprofloxacin against this strain.

It is important to note that the antimicrobial activity of EOs is highly dependent on their chemotype and the context of application. While our retail sample, characterized by an α-thujone/camphor-rich profile for SO, exhibited weak direct activity against *E. coli* under standardized in vitro conditions, recent studies have demonstrated that camphor/1,8-cineole-rich chemotypes of EO from SO can effectively reduce *E. coli* and *Salmonella enteritidis* populations in soil, seeds, and cilantro plants over extended periods [76]. These findings underscore the importance of chemotypic characterization and context-specific evaluation, when assessing the antimicrobial potential of SO-EO. The weak in vitro activity observed in our study should not be interpreted as a universal lack of efficacy but rather as a reflection of the specific chemotype and standardized testing conditions employed.

It is important to note that all 91 volatile compounds identified in the present study have been previously reported in the literature for the respective species. Therefore, the novelty of this work does not lie in the discovery of novel metabolites, but rather in the comparative, multi-genus quality assessment of commercial retail products, the verification of their phytochemical authenticity against ISO and pharmacopeial standards, and the integration of chemometric clustering with quantitative antimicrobial and antibiofilm data to establish chemotype-activity relationships in products that consumers actually use.

Taken together, these findings confirm that the antimicrobial and antibiofilm efficacy of the tested commercial Lamiaceae EOs is governed by functional group profile rather than botanical origin alone, with phenol-rich oils showing broad-spectrum potency and non-phenolic oils displaying selective activity. The functional group–level mechanistic interpretation provided here, linking phenolic hydroxyls, hydrocarbon precursors, ketones, and aldehydes to distinct membrane interactions, offers a level of structure–activity resolution that is uncommon in EO profiling studies and supports the reliability of pharmacy-distributed products when quality is assured.

## 4. Materials and Methods

### 4.1. EO Samples

Retail samples of the EOs were purchased as sealed, commercially packaged products from randomly selected pharmacies in Ankara, Türkiye, in 2025 and kept at +4 °C in a refrigerator prior to the experiments. One product per species was analyzed, representing six distinct commercial brands. All products were labeled as 100% pure EOs. No plant materials were collected, and no laboratory-scale extraction was performed by the authors; the study was designed to evaluate finished commercial products as they are available to consumers. The samples were stored at +4 °C in a refrigerator prior to analysis.

The commercial brand names of the tested EOs are not disclosed due to legal and commercial confidentiality constraints. However, full product identity, batch numbers, and supplier details have been documented and can be made available to the Editorial Office upon confidential request.

### 4.2. GC and GC-MS Analysis Method of the EO Samples

#### 4.2.1. GC-MS Analysis

The GC-MS analysis was carried out with an Agilent 5975 GC-MSD system (Agilent Technologies, Santa Clara, CA, USA). Innowax FSC column (60 m × 0.25 mm, 0.25 μm film thickness, Agilent Technologies, Santa Clara, CA, USA) was used with helium as the carrier gas (0.8 mL/min). GC oven temperature was kept at 60 °C for 10 min and programmed to 220 °C at a rate of 4 °C/min, and kept constant at 220 °C for 10 min and then programmed to 240 °C at a rate of 1 °C/min. The split ratio was adjusted to 40:1. The injector temperature was set at 250 °C. MS was recorded at 70 eV. The mass range was from *m*/*z* 35 to 450.

#### 4.2.2. GC Analysis

The GC analysis was carried out using an Agilent 6890N GC system (Agilent Technologies, Santa Clara, CA, USA). FID detector temperature was 300 °C. To obtain the same elution order as with GC-MS, simultaneous auto-injection was performed on a duplicate of the same column applying the same operational conditions. Relative percentage amounts of the separated compounds were calculated from FID chromatograms. The analysis results are presented in Table 1. Identification of the EO components was carried out by comparison of their relative retention times with those of authentic samples or by comparison of their relative retention index (RRI) to a series of *n*-alkanes (C_8_–C_40_), the standard mixture for retention index calculation (Sigma-Aldrich, St. Louis, MO, USA). The *n*-alkanes (C_8_–C_40_) were used solely as reference compounds for the calculation of relative retention indices (RRIs) and were not used for compound identification. A total of 52 compounds were definitively identified by comparison of their relative retention times (tR) with those of authentic reference standards analyzed under identical GC-MS conditions on the same HP-Innowax column. The remaining 39 compounds were identified by computer matching of their mass spectra against the Wiley GC/MS Library and MassFinder 4.0, supported by comparison of their relative retention indices (RRIs) with literature data and the in-house “Başer Library of Essential Oil Constituents [77,78]. No internal standard was used in the GC-MS analysis. Therefore, the reported data should be interpreted primarily as qualitative or semi-quantitative information. Relative percentage amounts were calculated from FID peak areas without correction for response factors and thus represent relative composition rather than absolute concentrations.

### 4.3. Antimicrobial Activity Assessment of the EO Samples

The antimicrobial capacity of the EO samples from Lamiaceae was evaluated against *P. aeruginosa* PAO1, *E. coli* ATCC 25922, *E. faecalis* ATCC 29212, *S. aureus* ATCC 29213, and *C. albicans* ATCC 10231 using the disc diffusion and broth microdilution methods. The assays were conducted according to the Kirby–Bauer method [79] and the relevant Clinical and Laboratory Standards Institute (CLSI) guidelines [80], respectively. The bacterial strains were subcultured on Mueller–Hinton agar (MHA, Merck, Darmstadt, Germany) and incubated at 37 °C for 18–24 h. *C. albicans* was subcultured on Sabouraud dextrose agar (SDA, Merck, Darmstadt, Germany) and incubated at 37 °C for 24–48 h. Fresh cultures were used in all experiments. Microbial inocula were prepared by suspending in phosphate-buffered saline (PBS, Sigma-Aldrich, St. Louis, MO, USA). The turbidity of each suspension was adjusted to a 0.5 McFarland standard using a densitometer (Grant Bio DEN-1, Ankara, Türkiye).

#### 4.3.1. Disc Diffusion Assay

To assess the disc diffusion assay, 100 µL of each microbial suspension was spread uniformly over the surface of the appropriate agar medium. MHA was used for the bacterial strains, whereas SDA was used for *C. albicans*. Sterile blank paper discs (6 mm, Bioanalyse, Ankara, Türkiye) were placed on the inoculated agar surfaces, and each disc was impregnated with 20 µL of the respective EO. The EOs were tested individually under identical experimental conditions. The plates were incubated at 37 °C for 18–24 h, while those inoculated with *C. albicans* were incubated at 37 °C for 24–48 h. Following incubation, the diameters of the inhibition zones were measured, and a clear halo zone was considered an inhibition zone.

Ciprofloxacin (5 µg/disc) (≥98% purity, Sigma-Aldrich, St. Louis, MO, USA) was used as the positive control for *P. aeruginosa* and *E. coli*. Ampicillin (10 µg/disc) (≥98% purity, Sigma-Aldrich, St. Louis, MO, USA) and rifampin (5 µg/disc) (≥95% purity, Sigma-Aldrich, St. Louis, MO, USA) were used as positive controls for *E. faecalis* and *S. aureus*, respectively. Amphotericin B (100 μg/disc) (≥80% purity, Sigma-Aldrich, St. Louis, MO, USA) was used as the positive control for *C. albicans*. Discs loaded with the solvent used to prepare the EO samples served as negative controls.

#### 4.3.2. MIC Determination

The MIC values of each EO were determined using a broth microdilution assay performed in sterile 96-well microplates. The procedure was adapted from the relevant CLSI recommendations, with Sabouraud dextrose broth (SDB, Merck, Darmstadt, Germany) used as the test medium for *C. albicans*. EO stock solutions were prepared in dimethyl sulfoxide (DMSO, ≥99.9% purity, Sigma-Aldrich, St. Louis, MO, USA) and serially diluted two-fold in the appropriate broth medium. Mueller–Hinton broth (MHB, Merck, Darmstadt, Germany) was used for the bacterial strains, whereas SDB was used for *C. albicans*. The final DMSO concentration in the wells did not exceed 1% (*v*/*v*) and exhibited no detectable effect on microbial growth. The diluted EO preparations were inoculated with the corresponding standardized microbial suspensions. Each microplate included growth-control wells containing broth and inoculum, sterility-control wells containing broth alone, and solvent-control wells containing broth, inoculum, and DMSO at the concentration used in the test wells. Plates containing the bacterial strains were incubated at 37 °C for 18–24 h, while those containing *C. albicans* were incubated at 35–37 °C for 24–48 h. The MIC was recorded as the lowest EO concentration at which no visible microbial growth was observed relative to the corresponding growth control. All disc diffusion and broth microdilution assays were performed in triplicate.

#### 4.3.3. Biofilm Inhibition Assay

The effect of the EO samples on biofilm formation was evaluated using a crystal violet (Sigma-Aldrich, St. Louis, MO, USA) microtiter plate assay. Fresh microbial cultures were adjusted to 0.5 McFarland and diluted in the appropriate growth medium to obtain the working inoculum. Aliquots of the microbial suspensions were dispensed into sterile, flat-bottomed 96-well plates and exposed to each EO at Sub-MIC (which is ½ MIC). Wells containing the microbial suspension without EO served as the untreated biofilm controls. The plates were incubated under static conditions at 37 °C for 24 h. After incubation, the contents of the wells were gently removed, and the wells were washed three times with sterile PBS to remove non-adherent cells. The attached biofilms were then fixed with methanol (analytical grade, ≥99.8% (Merck, Darmstadt, Germany) for 15 min and allowed to air-dry. Each well was stained with 0.1% (*w*/*v*) crystal violet for 30 min. The excess stain was subsequently removed by washing with distilled water (Milli-Q system, Millipore, Burlington, MA, USA). After the plates had dried, the retained crystal violet was solubilized with 30% acetic acid (≥99.7%, Merck, Darmstadt, Germany), and absorbance was measured at 595 nm using a microplate reader (BMG Labtech, CLARIOstar, Ortenberg, Germany). The biofilm inhibition assay experiments were conducted in triplicate, and the results are presented as mean ± standard deviation (SD).

The percentage of biofilm inhibition was calculated as follows:

Biofilm inhibition (%) = [(OD_595control_ − OD_595 sample_)/OD_595control_] × 100

#### 4.3.4. Statistical Analysis

Disc diffusion and biofilm inhibition data were analyzed separately using ordinary two-way analysis of variance. EO type and microorganism were included as independent factors, together with the interaction between them. The EOs were compared separately within each microorganism using Tukey’s multiple-comparisons test. Adjusted *p*-values were used for all pairwise comparisons. Data are expressed as mean ± standard deviation (SD) from three independent experiments. Statistical analyses were performed using GraphPad Prism version 10.3.1 (GraphPad Software, Boston, MA, USA), and differences were considered statistically significant at *p* < 0.05.

### 4.4. Chemometric and Multivariate Analysis

Unsupervised multivariate statistical analyses, including PCA and HCA integrated with heatmap visualization, were performed using the ClustVis web-based bioinformatic platform (http://biit.cs.ut.ee/clustvis/) (accessed on 19 August 2026). The selection of compounds for HCA and heatmap generation was based on an abundance threshold (≥3.0% in at least one EO sample) rather than on their frequency of occurrence across species; therefore, compounds unique to a single species were retained as discriminatory markers, while shared compounds defined common chemotypic features. The input matrix consisted of the relative percentage concentrations of the major volatile constituents (≥3.0 in at least one EO) identified across the six retail Lamiaceae EOs. Row scaling was applied using the unit variance (UV) scaling method to standardize the variance of all volatile compounds to unity. Principal components were calculated via singular value decomposition (SVD) with imputation. For HCA and heatmap generation, Euclidean distance was chosen as the distance metric for both rows (compounds) and columns (EO samples), and clustering was computed using Ward’s minimum variance linkage method. Qualitative sample grouping (Bioactivity_Group) was incorporated as an annotation track above the heatmap to evaluate chemotypic clustering in relation to biological potency.

## 5. Conclusions

The current study highlights the critical necessity of rigorous chemical and biological quality verification for off-the-shelf commercial EOs. By demonstrating that retail Lamiaceae EOs procured from regulated pharmacies maintain high phytochemical integrity and conform to ISO and pharmacopeial standards, our findings bridge a vital gap between commercial availability and therapeutic reliability. The integration of GC-MS profiling with chemometric clustering successfully demonstrates that their therapeutic efficacy is inextricably linked to its specific functional group profile and chemotype, providing a reliable framework for quality control and authentication. Furthermore, the exceptional broad-spectrum and anti-biofilm potency observed in phenol-dominant oils underscores their promise as natural alternatives in combating antimicrobial resistance, while the quantified neurotoxicity risk associated with high α-thujone sage oil emphasizes the indispensable role of strict dosing and regulatory oversight. Ultimately, procuring EOs through legally monitored distribution networks safeguards consumer safety and ensures predictable bioactivity outcomes.

Overall, the findings demonstrate that off-the-shelf commercial Lamiaceae EOs retain their expected chemotypic integrity and phytochemical profiles, underpinning their reliable therapeutic and consumer applications when quality is assured. Future research should expand this analytical framework to encompass larger market samples across diverse geographical batches, investigate synergistic interactions with conventional therapeutics, and explore advanced delivery systems to maximize the clinical translation of plant-derived volatiles in aromatherapy.

## Figures and Tables

**Figure 1:**
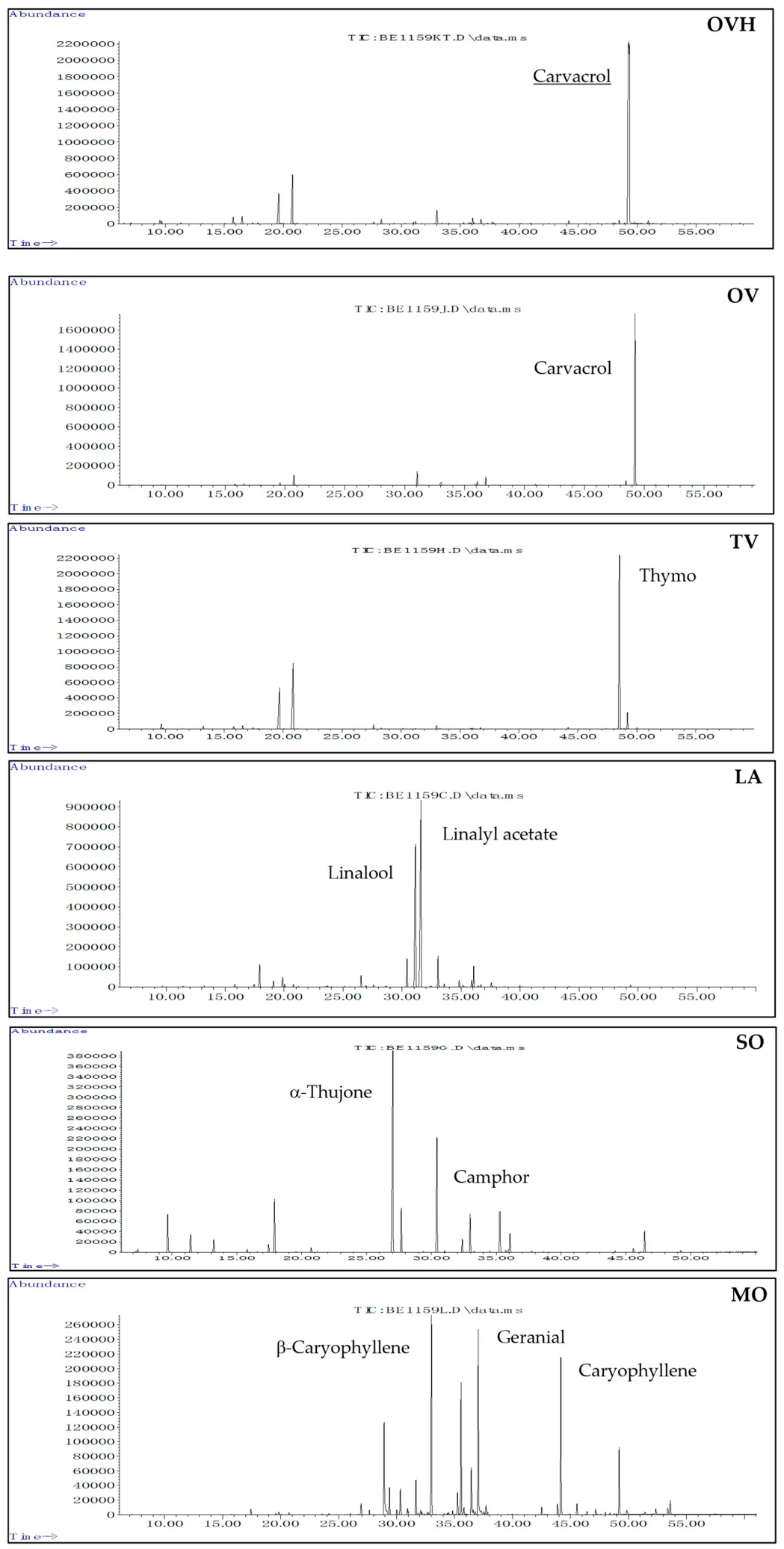
Total ion chromatograms (TICs) via GC-MS analyses of the six commercial Lamiaceae EOs. OVH: *Origanum vulgare* subsp. *hirtum*, OV: *Origanum vulgare*, TV: *Thymus vulgaris*, LA: *Lavandula angustifolia*, SO: *Salvia officinalis*, and MO: *Melissa officinalis*. The major characteristic peaks are annotated with their corresponding compound names.

**Figure 2:**
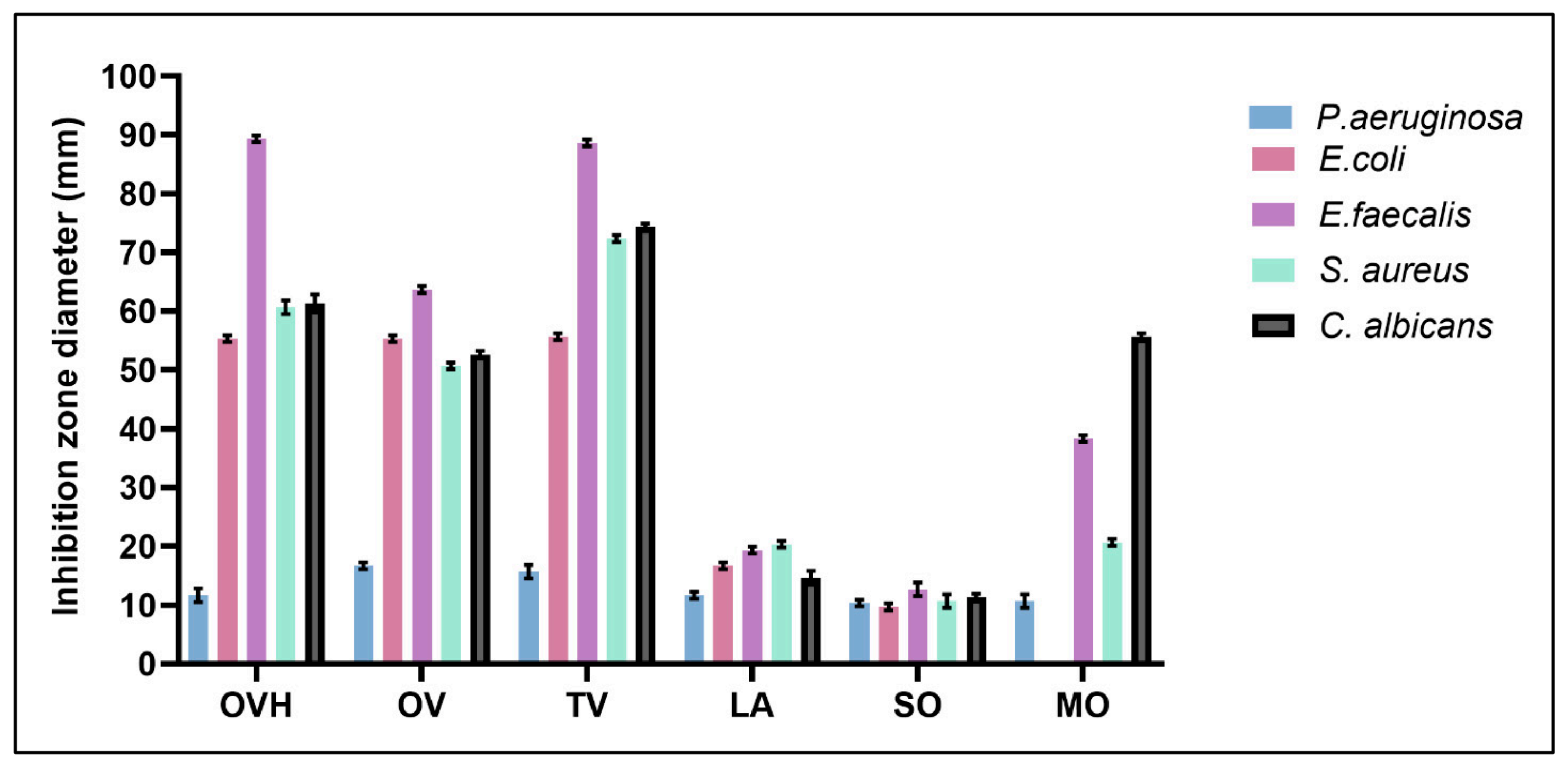
Inhibition zones produced by the EO samples against the test microorganisms, as determined using the disc diffusion method. Data are presented as mean ± SD. The effects of essential oil type, microbial strain, and their interaction were evaluated using two-way ANOVA (*p* < 0.0001).

**Figure 3:**
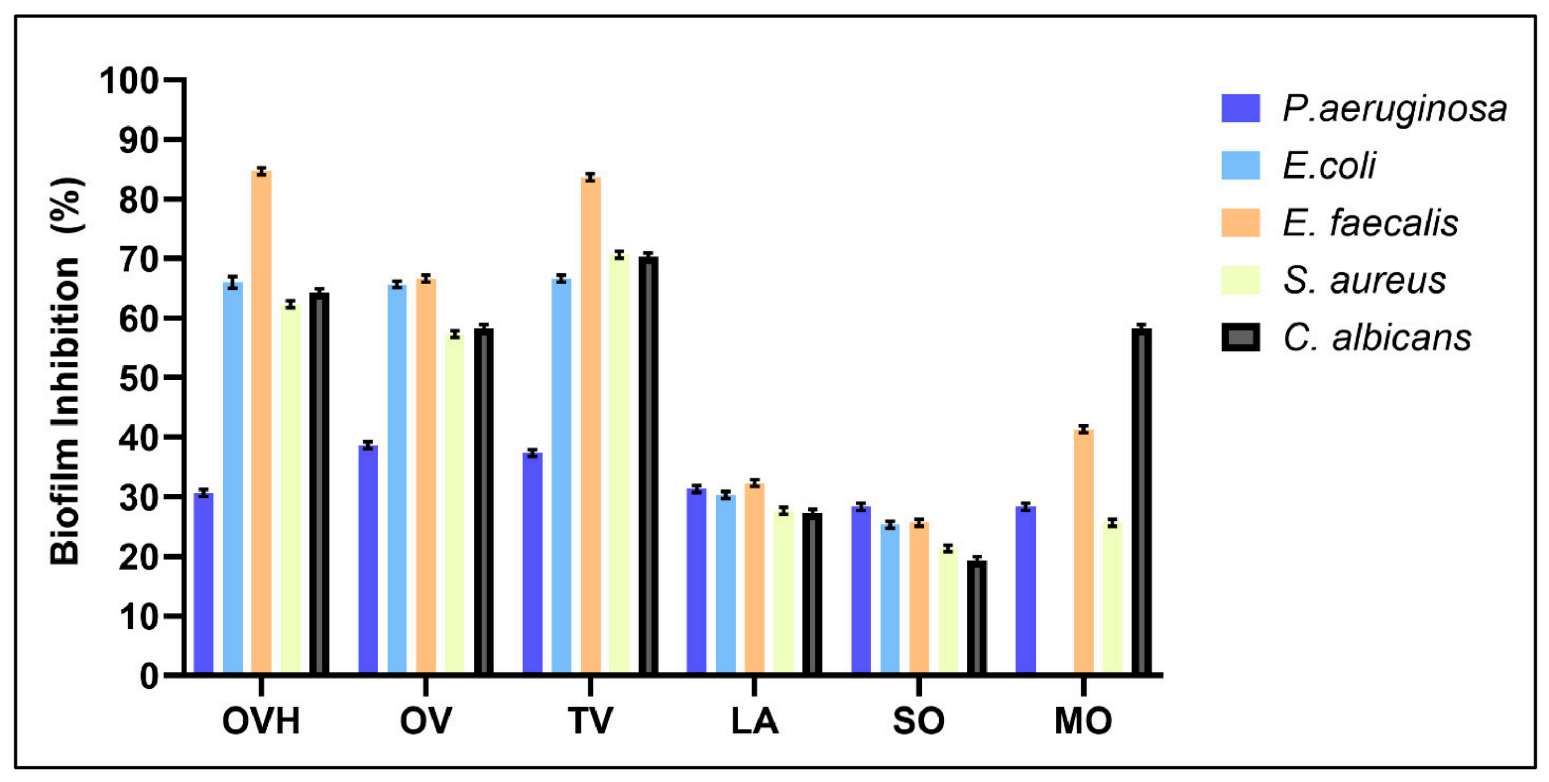
Inhibitory effects of the essential oils on biofilm formation by *P. aeruginosa*, *E. coli*, *E. faecalis*, *S. aureus*, and *C. albicans*. Biofilm inhibition is expressed as a percentage and data are presented as mean ± SD.

**Figure 4:**
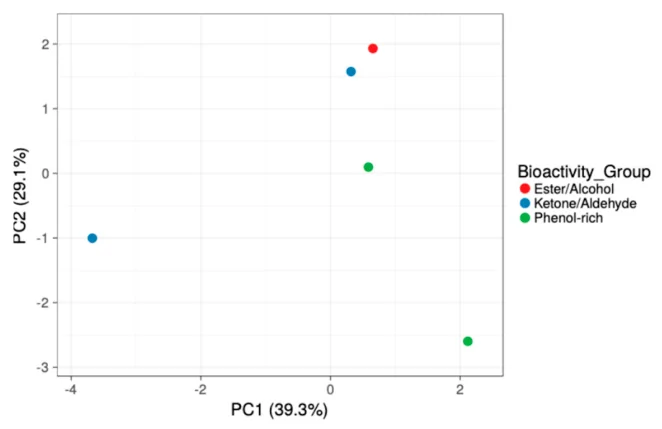
Principal component analysis (PCA) score plot of the selected retail Lamiaceae EOs based on their major volatile constituents (≥3.0%), displaying chemotypic clustering and prediction ellipses (95% confidence level).

**Figure 5:**
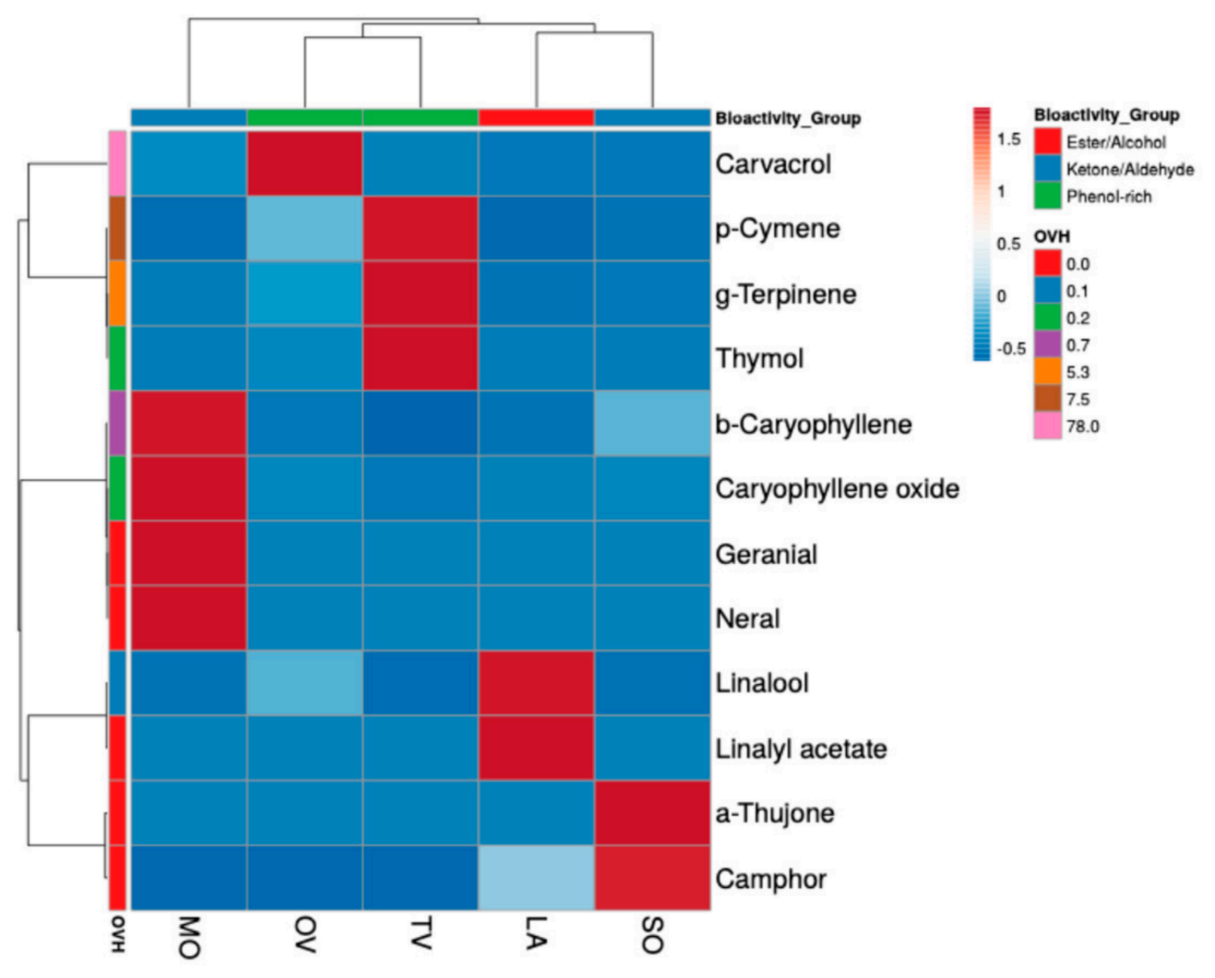
Two-way hierarchical cluster analysis (HCA) heatmap of major volatile constituents (≥3.0%) and retail Lamiaceae EO samples, calculated using Euclidean distance and Ward’s linkage method with bioactivity group annotation.

**Table 1:** Chemical composition (%) of selected retail essential oils from Lamiaceae.

RRI ^a^		OVH	OV	TV	LA	SO	MO	IM ^d^
1032	α-Pinene	0.5	0.4	2.0	0.2	6.8	0.2	t_R_, MS
1035	α-Thujene	0.4	- ^b^	0.3	-	0.2	-	t_R_, MS
1076	Camphene	0.3	0.2	tr	0.2	3.7	0.1	t_R_, MS
1118	β-Pinene	0.1	-	1.2	0.2	2.6	0.1	t_R_, MS
1132	Sabinene	-	-	-	0.1	tr	-	t_R_, MS
1174	Myrcene	0.9	0.8	0.9	0.5	0.7	0.2	t_R_, MS
1176	α-Phellandrene	0.1	0.1	0.1	-	-	-	t_R_, MS
1188	α-Terpinene	1.0	0.7	1.0	-	0.1	-	t_R_, MS
1203	Limonene	0.2	0.2	0.5	0.6	1.5	0.6	t_R_, MS
1213	1,8-Cineole	-	-	-	3.9	8.0	0.2	t_R_, MS
1218	β-Phellandrene	0.2	0.2	0.3	-	-	-	t_R_, MS
1246	(*Z*)-β-Ocimene	-	-	-	1.0	-	Tr ^c^	MS
1255	γ-Terpinene	5.3	1.5	16.7	-	0.2	0.3	t_R_, MS
1265	3-Octanone	-	-	0.1	0.6	-	-	MS
1266	(*E*)-β-Ocimene	-	-	-	1.7	-	0.3	MS
1280	*p*-Cymene	7.5	5.1	24.2	0.1	0.7	0.3	t_R_, MS
1282	Hexyl acetate	-	-	-	0.5	-	-	t_R_, MS
1290	Terpinolene	0.1	0.1	tr	0.1	0.2	-	t_R_, MS
1345	3-Octyl acetate	-	-	-	0.1	-	-	MS
1350	Hexyl propionate	-	-	-	0.1	-	-	MS
1353	Hexyl isobutyrate	-	-	-	0.2	-	-	MS
1360	Hexanol	-	-	-	0.1	-	-	t_R_, MS
1386	1-Octenyl acetate	-	-	-	0.1	-	-	MS
1393	3-Octanol	-	-	-	0.1	-	-	MS
1424	Hexyl butyrate	-	-	-	1.3	-	-	MS
1437	α-Thujone	-	-	-	-	33.8	-	t_R_, MS
1438	Hexyl 2-methyl butyrate	-	-	-	0.2	-	-	MS
1450	*trans*-Linalool oxide (*Furanoid*)	-	-	-	0.1	-	-	MS
1451	β-Thujone	-	-	-	-	5.8	-	t_R_, MS
1452	1-Octen-3-ol	0.2	0.2	0.9	-	-	0.3	MS
1474	*trans*-Sabinene hydrate	0.4	-	0.2	-	-	-	MS
1478	*cis*-Linalool oxide (*Furanoid*)	-	-	-	0.1	-	-	MS
1487	Citronellal	-	-	-	-	-	8.6	t_R_, MS
1497	α-Copaene	tr	-	-	-	0.2	1.9	t_R_, MS
1532	Camphor	-	-	-	3.8	15.0	-	t_R_, MS
1535	β-Bourbonene	tr	0.1	-	-	-	1.9	MS
1549	β-Cubebene	-	-	-	-	-	0.4	t_R_, MS
1553	Linalool	0.1	6.6	0.1	37.2	0.3	0.6	t_R_, MS
1556	*cis*-Sabinene hydrate	0.3	-	0.1	-	-	-	MS
1565	Linalyl acetate	-	-	-	36.1	-	-	t_R_, MS
1571	Methyl citronellate	-	-	-	-	-	2.0	t_R_, MS
1591	Bornyl acetate	-	-	-	-	1.4	-	t_R_, MS
1600	β-Elemene	-	-	-	-	-	tr	t_R_, MS
1611	Terpinen-4-ol	0.9	0.8	0.3	-	0.1	-	t_R_, MS
1612	β-Caryophyllene	0.7	1.4	0.5	1.3	3.8	16.7	t_R_, MS
1614	Carvacrol methyl ether	0.1	0.1	-	-	-	-	t_R_, MS
1616	Hotrienol	-	-	-	tr	-	-	MS
1617	Lavandulyl acetate	-	-	-	1.9	-	-	t_R_, MS
1628	Aromadendrene	-	-	0.1	-	-	-	MS
1631	Hexyl tiglate	-	-	-	0.3	-	-	t_R_, MS
1668	(*Z*)-β-Farnesene	-	-	-	0.9	-	tr	MS
1686	Lavandulol	-	-	-	0.3	-	-	t_R_, MS
1687	α-Humulene	0.1	-	0.1	-	7.1	2.2	t_R_, MS
1690	Cryptone	-	-	-	tr	-	-	MS
1694	Neral	-	-	-	-	-	11.3	t_R_, MS
1704	γ-Muurolene	0.1	-	-	-	0.3	-	MS
1704	(E)-Methyl geranate	-	-	-	-	-	0.9	t_R_, MS
1706	α-Terpineol	0.1	0.4	0.1	0.8	-	-	t_R_, MS
1708	Ledene	-	-	0.1	-	-	-	MS
1719	Borneol	0.5	1.5	0.2	2.6	2.2	-	t_R_, MS
1726	Germacrene D	-	-	-	-	-	3.6	t_R_, MS
1733	Neryl acetate	-	-	-	0.3	-	-	t_R_, MS
1737	(*Z*,*E*)-α-Farnesene	-	-	-	-	-	0.7	MS
1740	α-Muurolene	-	-	-	-	-	0.6	MS
1741	β-Bisabolene	0.3	3.1	0.2	-	-	-	t_R_, MS
1742	Geranial	-	-	-	-	-	18.3	t_R_, MS
1750	*trans*-Linalool oxide (*Pyranoid*)	-	-	-	-	-	-	MS
1765	Geranyl acetate	-	-	-	0.2	-	-	t_R_, MS
1770	*cis*-Linalool oxide (*Pyranoid*)	-	-	-	-	-	-	MS
1773	δ-Cadinene	0.3	0.1	-	-	0.3	0.7	MS
1776	γ-Cadinene	0.1	0.1	-	-	0.1	0.5	MS
1804	Myrtenol	-	-	-	-	0.2	-	t_R_, MS
1808	Nerol	-	-	-	tr	-	tr	t_R_, MS
1857	Geraniol	-	-	-	0.4	-	0.3	t_R_, MS
1864	*p*-Cymen-8-ol	tr	tr	tr	-	-	-	t_R_, MS
1890	Carvacryl acetate	0.1	0.2	-	-	-	-	t_R_, MS
1949	(Z)-3-Hexenyl nonanoate	-	-	-	tr	-	-	MS
2001	Isocaryophyllene oxide	-	-	-	-	-	0.9	MS
2008	Caryophyllene oxide	0.2	0.3	-	0.2	0.3	12.7	t_R_, MS
2071	Humulene epoxide-II	-	-	-	-	0.5	1.0	MS
2104	Viridiflorol	-	-	-	-	2.1	0.4	MS
2131	Hexahydrofarnesyl acetone	-	-	-	-	-	0.4	MS
2181	Isothymol	tr	tr	tr	-	-	-	MS
2198	Thymol	0.2	1.3	48.1	-	-	-	t_R_, MS
2221	Isocarvacrol	tr	0.2	tr	-	-	-	MS
2239	Carvacrol	78.0	72.6	1.6	-	-	4.0	t_R_, MS
2256	*epi*-α-Bisabolol	-	-	-	0.3	-	-	MS
2260	Citronellic acid	-	-	-	-	-	0.7	t_R_, MS
2392	Caryophyllenol II	-	-	-	-	-	1.0	MS
2679	Manool	-	-	-	-	0.7	-	MS
	Total	99.3	98.3	99.9	98.7	98.9	90.6	

^a^ Relative retention indices (RRI) calculated against *n*-alkanes ^b^ Not detected ^c^ Trace (<0.1%) ^d^ Identification method; t_R_, identification based on the retention times of authentic standards analyzed under identical conditions on the HP Innowax column (52 compounds); MS, identification based on computer matching of the MS with the Wiley and MassFinder libraries and comparison with literature data (39 compounds).

**Table 2:** Relative distribution (%) of chemical functional classes detected in the selected retail Lamiaceae EO samples.

Chemical Class	Relative Distribution (%)					
	OVH	OV	TV	LA	SO	MO
Total Alcohol	1.8	9.3	0.6	40.9	2.5	0.9
Total Phenol	78.2	73.9	49.7	0.0	0.0	4.0
Total Aldehyde/Ketone	0.0	0.0	0.0	3.8	54.6	38.2
Total Ester	0.1	0.2	0.1	39.5	1.4	2.9
Monoterpene Hydrocarbons	16.3	10.6	47.3	4.4	16.6	8.8
Sesquiterpene Hydrocarbons	1.6	4.8	0.9	2.2	11.7	28.0
Total Oxide/Ether	0.1	0.1	0.0	8.3	8.8	14.6

**Table 3:** Inhibition zones (mm) of antibacterial and antifungal results of the EO samples using the disc diffusion method.

EO Samples	*P. aeruginosa* PAO1	*E. coli* ATCC 25922	*E. faecalis* ATCC 29212	*S. aureus* ATCC 29213	*C. albicans* ATCC 10231
OVH	11.67 ± 1.15	55.33 ± 0.58	89.33 ± 0.58	60.67 ± 1.15	61.33 ± 1.53
OV	16.67 ± 0.58	55.33 ± 0.58	63.67 ± 0.58	50.67 ± 0.58	52.67 ± 0.58
TV	15.67 ± 1.15	55.67 ± 0.58	88.67 ± 0.58	72.33 ± 0.58	74.33 ± 0.58
LA	11.67 ± 0.58	16.67 ± 0.58	19.33 ± 0.58	20.33 ± 0.58	14.67 ± 1.15
SO	10.33 ± 0.58	9.67 ± 0.58	12.67 ± 1.15	10.67 ± 1.15	11.33 ± 0.58
MO	10.67 ± 1.15	0.00 ± 0.00	38.33 ± 0.58	20.66 ± 0.58	55.67 ± 0.58
Positive controls *	50.33 ± 0.58	34.33 ± 0.58	48.33 ± 0.58	45.33 ± 0.58	22.67 ± 0.58

* Values are expressed as mean ± SD. The reference antimicrobial agents were ciprofloxacin for *P. aeruginosa* PAO1 and *E. coli* ATCC 25922, ampicillin for *E. faecalis* ATCC 29212, rifampin for *S. aureus* ATCC 29213, and amphotericin B for *C. albicans* ATCC 10231.

**Table 4:** MIC values of the EO samples against the test microorganisms (% *v*/*v*).

EO Samples	*P. aeruginosa* PAO1	*E. coli* ATCC 25922	*E. faecalis* ATCC 29212	*S. aureus* ATCC 29213	*C. albicans* ATCC 10231
OVH	6.25	1.56	0.39	1.56	1.56
OV	3.12	1.56	0.78	1.56	1.56
TV	3.12	1.56	0.19	0.78	0.78
LA	6.25	3.12	3.12	3.12	6.25
SO	6.25	6.25	6.25	6.25	6.25
MO	6.25	ND *	3.12	6.25	1.56

* No inhibitory activity detected within the tested concentration range.

**Table 5:** Biofilm inhibition (%) of the EO samples against the test microorganisms.

EO Samples	Biofilm Inhibition (%) * ± SD				
	*P. aeruginosa* PAO1	*E. coli* ATCC 25922	*E. faecalis* ATCC 29212	*S. aureus* ATCC 29213	*C. albicans* ATCC 10231
OVH	30.67 ± 0.58	66.00 ± 1.00	84.67 ± 0.58	62.33 ± 0.58	64.33 ± 0.58
OV	38.67 ± 0.58	65.67 ± 0.58	66.67 ± 0.58	57.33 ± 0.58	58.33 ± 0.58
TV	37.33 ± 0.58	66.67 ± 0.58	83.67 ± 0.58	70.67 ± 0.58	70.33 ± 0.58
LA	31.33 ± 0.58	30.33 ± 0.58	32.33 ± 0.58	27.67 ± 0.58	27.33 ± 0.58
SO	28.33 ± 0.58	25.33 ± 0.58	25.67 ± 0.58	21.33 ± 0.58	19.33 ± 0.58
MO	28.33 ± 0.58	0.00 ± 0.00	41.33 ± 0.58	25.67 ± 0.58	58.33 ± 0.58

* Values are presented as biofilm inhibition (%, mean) ± standard deviation (SD) (*n* = 3).

## Data Availability

The data presented in this study are available in the article. Further inquiries can be directed to the corresponding author.

## Abbreviations

The following abbreviations are used in this manuscript:

ADI	Acceptable daily intake
ANOVA	Analysis of variance
ATCC	American type of culture collection
ATP	Adenosine triphosphate
CFU	Colony forming unit
CLSI	Clinical and Laboratory Standards Institute
DMSO	Dimethyl sulfoxide
EMA	European Medicine Agency
EO	Essential oil
EPS	Extracellular polymeric substance
FID	Flame ion detector
FT-IR	Fourier transform-infrared spectroscopy
GABA_A_	Gamma-aminobutyric acid type A
GC-MS	Gas chromatography-mass spectrometry
HCA	Hierarchical cluster analysis
ISO	International Organization for Standardization
LA	*Lavandula angustifolia*
MHA	Mueller–Hinton agar
MHB	Mueller–Hinton broth
MIC	Minimum inhibitory concentration
MO	*Melissa officinalis*
ND	Not detected
NG	No grow
OV	*Origanum vulgare*
OVH	*Origanum vulgare* subsp. *hirtum*
PCA	Principal component analysis
qNMR	Quantitative nuclear magnetic resonance spectroscopy
PBS	Phosphate-buffered saline
RRI	Relative retention index
SD	Standard deviation
SDA	Sabouraud dextrose agar
SDB	Sabouraud dextrose broth
SO	*Salvia officinalis*
SVD	Singular value decomposition
TIC	Total ion chromatography
tR	Retention time
TV	*Thymus vulgaris*
UV	Unit variance
